# Database Mining to Unravel the Ecology of the Phylum Chloroflexi in Methanogenic Full Scale Bioreactors

**DOI:** 10.3389/fmicb.2020.603234

**Published:** 2021-01-20

**Authors:** Patricia Bovio-Winkler, Angela Cabezas, Claudia Etchebehere

**Affiliations:** ^1^Microbial Ecology Laboratory, Department of Microbial Biochemistry and Genomic, Biological Research Institute “Clemente Estable,” Montevideo, Uruguay; ^2^Instituto Tecnológico Regional Centro Sur, Universidad Tecnológica, Durazno, Uruguay

**Keywords:** Chloroflexi, full-scale methanogenic reactors, wastewater, filamentous bacteria, 16S rRNA amplicon sequence, meta-analysis

## Abstract

Although microbial communities of anaerobic bioreactors have been extensively studied using DNA-based tools, there are still several knowledge gaps regarding the microbiology of the process, in particular integration of all generated data is still limited. One understudied core phylum within anaerobic bioreactors is the phylum Chloroflexi, despite being one of the most abundant groups in anaerobic reactors. In order to address the abundance, diversity and phylogeny of this group in full-scale methanogenic reactors globally distributed, a compilation of 16S ribosomal RNA gene sequence data from 62 full-scale methanogenic reactors studied worldwide, fed either with wastewater treatment anaerobic reactors (WTARs) or solid-waste treatment anaerobic reactors (STARs), was performed. One of the barriers to overcome was comparing data generated using different primer sets and different sequencing platforms. The sequence analysis revealed that the average abundance of Chloroflexi in WTARs was higher than in STARs. Four genera belonging to the Anaerolineae class dominated both WTARs and STARs but the core populations were different. According to the phylogenetic analysis, most of the sequences formed clusters with no cultured representatives. The Anaerolineae class was more abundant in reactors with granular biomass than in reactors with disperse biomass supporting the hypothesis that Anaerolineae play an important role in granule formation and structure due to their filamentous morphology. Cross-study comparisons can be fruitfully used to understand the complexity of the anaerobic digestion process. However, more efforts are needed to standardize protocols and report metadata information.

## Introduction

Anaerobic digestion is an efficient biological process widely applied to treat solid organic waste and wastewater, where the organic matter can be converted into a renewable energy source known as biogas. As it is a mixture containing mainly methane (CH_4_) and carbon dioxide (CO_2_), it can be used as a replacement for fossil fuels to generate heat or electricity (Angenent et al., 2004; Verstraete et al., 2005; Appels et al., 2011).

Chloroflexi has been reported as one of the most predominant phylum present in solid waste and wastewater treatment systems (Shu et al., 2015; Petriglieri et al., 2018; Bovio et al., 2019). In particular, the Anaerolineae class has been identified as one of the core microbial populations in full-scale anaerobic reactors (Nelson et al., 2011; St-Pierre and Wright, 2014; Bovio et al., 2019). From 12 isolated strains within the Anaerolineae class, five have been isolated from wastewater treatment anaerobic reactors (WTARs) and one from solid-waste treatment anaerobic reactors (STARs) (Sekiguchi et al., 2003; Yamada et al., 2006, 2007a; Sun et al., 2016). All isolated species share similar phenotypic traits such as filamentous morphology, strict anaerobic growth, and the ability to ferment carbohydrates or amino acids. However, the ecological role of the Anaerolineae remains uncertain due to the scarcity of isolates and annotated genome sequences.

In recent years, efforts to assemble genomes from metagenomes have increased due to the difficulty to obtain Chloroflexi strains in pure culture. Genomes belonging to Anaerolineales (*Candidatus* Brevefilum fermentans CAMBI-1), Ardenticatenales (*Candidatus* Promineofilum), and Caldilineales (*Candidatus* Amarolinea aalborgensis) orders have been assembled from shotgun metagenomic sequencing data obtained from aerobic and anaerobic reactors (McIlroy et al., 2017, 2016; Andersen et al., 2018). These genomes showed similar potential metabolism compared to isolated members. It has been suggested that the common prevalence of Anaerolineae class in WTARs and STARs might be due to their advantageous cellular adhesiveness, their potential as cellulose degraders and/or because of being anaerobic syntrophs (Xia et al., 2016). For example, some Anaerolineae species require syntrophic association with hydrogenotrophic methanogens for efficient growth (Sekiguchi et al., 2001; Yamada et al., 2005, 2006).

The two most common types of anaerobic digestion systems currently in use are up-flow anaerobic sludge blanket (UASB) reactors and continuously stirred tank reactors (CSTR). In UASB systems, and variations such as expanded granular sludge bed (EGSB) and internal circulation (IC) reactors, the active microbial biomass form compact anaerobic granules which settle against the hydraulic up-flow inside the reactor, preventing biomass washout (Skiadas et al., 2003). Contrastingly, in CSTR systems, generally used for solid waste anaerobic treatment, the active microbial biomass typically does not exhibit granulation but grow in suspension and is constantly removed from the system (Hofman-Bang et al., 2003; Klocke et al., 2007). It has been hypothesized that Chloroflexi are relevant for the granule skeleton formation in WTARs as they grow as filaments and therefore play an important role in sludge sedimentation (Yamada et al., 2005). On the other hand, Chloroflexi has been reported to be occasionally involved in bulking episodes in WTARs, caused by their overgrowth, generating biomass washout (Sekiguchi et al., 2001, 2015; Yamada et al., 2007b; Borzacconi et al., 2008; Li et al., 2008). Meanwhile, it has been suggested that, in STARs some of these Chloroflexi derive and migrate from the aerobic activated sludge community when they are coupled together, although many were identified as being exclusive to the digester (Kirkegaard et al., 2017; Petriglieri et al., 2018).

The phylum Chloroflexi has been studied in STARs and WTARs mainly by molecular methods in separate studies. However, a comparison of Chloroflexi diversity and abundance between full scale STARs and WTARs has still not been investigated. During the last decade, the amount of studies using amplicon sequencing to analyze the microbial community in STARs and WTARs have been in constant growth, but the experimental approaches or data analysis differ widely. This strongly limits our ability to compare among studies and draw general conclusions regarding their diversity or the identification of important taxa. The recent ecosystem-specific Microbial Database for Activated Sludge (MiDAS) 16S rRNA gene amplicon sequencing-based survey, facilitates the understanding of wastewater treatment ecosystem diversity and function (Nierychlo et al., 2020). Data analysis appears to be an important area where further improvements and unification of experimental procedures are necessary. The main objective of this study was to answer the following questions: Is there a particular Chloroflexi population predominant in all anaerobic reactors or are there several groups? Is there a selection of different groups between STARs and WTARs? To address these questions, we conducted a meta-analysis of publicly available microbial datasets generated by high-throughput sequencing in 62 full scale anaerobic reactors treating 29 different solid wastes or wastewaters.

## Materials and Methods

### Data Collection and Composition

This meta-analysis includes sequences from published papers, all of which used bacterial 16S rRNA gene sequencing to survey the microbial community in full-scale anaerobic reactors. Seventeen studies were selected that comprised 62 methanogenic reactors: 27 reactors treating wastewater (WTAR) and 35 reactors treating solid waste (STAR). The configuration of the reactors, operational data, substrates, sequencing platforms, and primer sets are summarized in Table 1 (Supplementary Table 1). PCR-generated amplicons from bacterial 16S rRNA genes were sequenced using a diversity of primer sets targeting different regions of the 16S rRNA gene of bacteria and archaea (Table 1 and Supplementary Table 1). The data sets were retrieved from the Sequence Read Archive (SRA) of the National Center for Biotechnology Information (NCBI, United States) or were requested from the authors directly. The sample metadata for the processing of the raw data were extracted from published articles or provided by the authors. Some other operational data such as temperature, chemical oxygen demand removal (COD), pH, hydraulic retention time (HTR) and volatile fatty acid (VFA) were also collected in this study, but these data were only present for some reactors. Accordingly, we did not include those variables in this meta-analysis.

**TABLE 1:** Studies included in this meta-analysis and operational parameters.

	Reactor configuration	Amount of reactors	Substrate	Temperature	Platform	Primers	Study
WTAR	UASB	4	Brewery	Mesophilic	Roche 454 FLX	27F-338R	Werner et al., 2011
	IC	1					
	EGSB	4					
	UBF	1	High-strength pharmaceutical		Illumina MiSeq	8F-338R	Ma et al., 2017
	UASB	2	Pulp and paper		Roche 454 GS-FLX	357F-926R	Shu et al., 2015
		1	Starch				
		1	Juice				
		2	Municipal and campus domestic sewage				
		1	Potato	NA	Illumina MiSeq	515F-806R	Zhu et al., 2017
		1	Poultry slaughterhouse		Ion 318^TM^	577F-924R	Delforno et al., 2016
		1	Vinasse	Mesophilic	Roche 454 FLX	515F-806R	Bovio et al., 2019
		3	Dairy				
		1	Malting		Ion Torrent PGM	520F-802R	
		1	Paper mill	NA	Illumina MiSeq	515F-806R	Sposob et al., 2018
	AnaEG	1	Cassava, potato, maize starch			341F-805R	Qin et al., 2018
	IC	1	Ethanol processing wastewater	Mesophilic			Qin et al., 2019
	AnaEG	1					
STAR	–	1	Municipal solid wastes		Roche 454	27F-518R	Cardinali-Rezende et al., 2016
	Plug-flow	2	Dairy cattle manure suppl. with cheese			27F-519R	St-Pierre and Wright, 2014
			Cattle manure with ice cream waste				
	Complete mix	1	Cattle manure with oil waste				
	CSTR	1	Food waste-recycling wastewater	Thermophilic		787F-1492R	Lee et al., 2016
		2	Primary and biological sewage sludge	Mesophilic	Ion Torrent PGM	515F-806R	Hao et al., 2016
		3	Mixed sludge		Illumina MiSeq	515F-805R	Liu et al., 2017
			Thin stillage				
			Agricultural waste				
		1	Maize silage		Roche 454 GS	27F-519R	Lucas et al., 2015
		7	Cattle manure		Illumina MiSeq	515F-806R	Li et al., 2015
	USR	1					
		1	Swine manure				
	CSTR	11					
		4	Sewage sludge		Roche 454 GS-FLX	787F-1492R	Shin et al., 2016

### Data Processing, Clustering, and Taxonomic Assignments

Raw reads (FASTQ) from each study were assessed for nucleotide quality and adaptors contamination with FastQC v0.11.5.^1^ The raw reads were processed through Trimmomatic v0.38 (Bolger et al., 2014) for removal of ambiguous reads, adapters, and low-quality sequences to obtain high quality reads (slidingwindow:4:25, minlen:200). Depending on the sequencing platform (single-end or paired-end) the pre-processed raw data was imported in QIIME2 v.2019.10 (Bolyen et al., 2019). For demultiplexing the raw data, qiime cutadapt demux-paired or demux-single plugins were used with default settings. All chimeras and borderline chimeras were detected and discarded using the qiime vsearch uchime-denovo plugin (vsearch v2.7.0) with defaults settings (Rognes et al., 2016).

### Analysis Strategy of the Sequencing Data

Given the heterogeneity of the selected hypervariable regions targeted within the 16S rRNA gene, we used closed-reference OTU picking method. In this method, the input sequences are clustered against a reference database as a means of being able to combine non-overlapping tags (Rideout et al., 2014). Because input sequences are not compared directly to one another (*de novo* OTU picking method), but rather to an external reference, the input sequences do not need to overlap. This is essential for this analysis because we are performing a meta-analysis including sequences derived from different amplification products of the same marker gene, such as the V1–V2 and V3–V4 regions of the 16S rRNA (e.g., as in the meta-analysis performed in Caporaso et al. (2010). As a result, for each short read we obtained a representative reference sequence of approximately 1500 bp, which enabled the comparison of non-overlapping tags. For this reason, it was not possible to use the ASV method in which overlapping sequences are needed. OTUs were then picked at 97% sequence identity against the SILVA 138 reference database using the plugin of vsearch cluster-features-closed-reference plugin (–p-perc-identity 0.97) (Quast et al., 2012; Glöckner et al., 2017). Taxonomy was assigned with qiime feature-classifier-sklearn (–p-confidence 0.8) plugin against the MiDAS 3.6 database (Nierychlo et al., 2020). MiDAS 3.6 database is a comprehensive ecosystem-specific reference database for activated sludge and anaerobic digesters which provides a taxonomic classification at all ranks for all sequences (Dueholm et al., 2019; Nierychlo et al., 2020). The singletons were deleted and each count table was then normalized to the lowest number of reads (561 reads per sample). Even though this sequence number is low, we decided to prioritize the inclusion of all samples and all variable regions amplified by the most frequently used primers. After the normalization, 10 reactors presented 0 Chloroflexi sequences which were the reactors with lowest relative abundance (below 0.5%) of Chloroflexi in the microbial community.

### Data Analyses and Visualization

As equal variances are a prerequisite of permutational multivariate analysis of variance (PERMANOVA) (Daniels et al., 2011), previous to this analysis we performed a variance homogeneity check (betadisper function of the “vegan” package) (Oksanen et al., 2013). In order to decipher the significant influence of explicative factors on the microbial diversity (e.g., reactor type, platform, target region, and substrate, Table 1), a PERMANOVA with adonis function, and principal coordinates analysis (PCoA) based on Bray–Curtis dissimilarity distance was calculated using R software version 3.5.1 (R Core Team, 2013) in R Studio environment Version 1.0.153. Correction for multiple hypothesis testing was performed with the Benjamini–Hochberg (BH) procedure using the p.adjust function in R (Benjamini and Hochberg, 1995). Two-way analysis of variance (ANOVA) performed with R was used to test the effect of reactor type, platform, target region, and substrate on the relative abundance of phylum Chloroflexi. The differences and correlations were considered significant at *p* < 0.05. In order to determine significant difference in the relative abundance of Chloroflexi between STAR and WTARs, the Shapiro–Wilk (to test normal distribution) and Kruskal–Wallis (to test equality of means) tests were applied in R. The correlation between the relative abundance of Euryarchaeota and Chloroflexi was performed with the Spearman’s rho correlation test in R.

The diversity indices (Shannon H, Evenness, and Chao-1), and the *t*-test and Kruskal–Wallis test (for normal and non-normal distribution, respectively) to test the equal means over diversity indexes was performed using past software (v4.02) (Hammer et al., 2001). Boxplots and PCoA plots were generated using R package ggplot2 (Wickham, 2017).

### Phylogenetic Tree

A phylogenetic tree was constructed to evaluate the phylogenetic diversity of the phylum Chloroflexi. It included the reference OTUs obtained with the closed-reference OTU picking method with a relative abundance greater than 5% in at least one sample, reference OTUs which were part of the core microbiome (defined as Chloroflexi members composed by OTUs shared by 50% of the STARs or WTARs) and 16S rRNA sequences from assembled genomes and cultured representatives from the phylum Chloroflexi. *Thermotoga* spp. sequence served as an outgroup for rooting the tree. The phylogenetic tree was performed using the maximum likelihood phylogenetic tree method with the generalized time reversible (GTR) (Nei and Kumar, 2000) substitution model and GAMMA distribution model using 1000 bootstraps in MEGA 10.0 (Kumar et al., 2018). The resultant phylogenetic trees were visualized in iTOL (Letunic and Bork, 2019).

### Primer Coverage Rate

The coverage rates for Chloroflexi populations of the different primer sets used can differ with the target location within the 16S rRNA gene. The Silva database Test Prime tool (Klindworth et al., 2013) was used to evaluate primer bias over Chloroflexi populations at different taxonomic levels with settings recommended in the Silva database Test Prime tutorial (1 mismatch and 5 bases).

## Results

In the present study, high-throughput 16S rRNA sequence data from 17 different studies were compiled, including 62 full-scale anaerobic reactors that treat 29 different types of wastewaters or solid wastes. The data retrieved was generated using different primer pairs, sequencing platforms and sequencing depth which required bioinformatics tools to be adapted in order to be able to compare the data properly. Moreover, the primer coverage for the target group was also determined and analyzed. Finally, the diversity, phylogeny, and abundance of the Chloroflexi population across these methanogenic reactors, applying closed-reference method, were determined.

### *In silico* Analysis of the Coverage Rate of Chloroflexi Primer Pairs

As different primer pairs were used to generate sequences in the different investigations, we analyzed how this might affect the recovery of Chloroflexi populations, at different taxonomic levels, frequently found in methanogenic reactors. The *in silico* analysis performed showed different coverage of the phylum when different primer pairs are used. The primer sets directed to the region between V3 and V5 hypervariable regions (47 reactors) showed the highest coverage for the Chloroflexi phylum (between 78 and 91%) (Table 2). Meanwhile, the coverage for families commonly found in methanogenic reactor as Anaerolineaceae, Ardenticatenaceae and Caldilineaceae was between 71 and 100%. The primer sets that covered hypervariable regions V1–V2 and V5–V9 (15 reactors) were the least efficient covering the Chloroflexi phylum (58–70%). For Anaerolineaceae, Ardenticatenaceae, and Caldilineaceae the coverage was between 61 and 100%, with the exception of V1–V2 region which showed a coverage of 5% for Anaerolineaceae (Table 2).

**TABLE 2:** *In silico* primer coverage prediction for the 10 primers sets used in the studies restricted to the phylum Chloroflexi in SILVA Test Prime analysis (SSU Ref 138 NR).

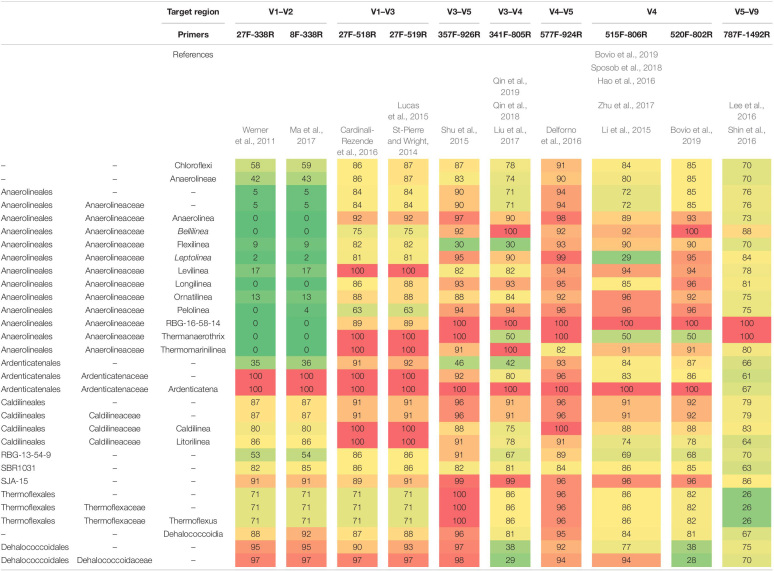

*Color red indicates higher primer coverage, while green color indicates lower primer coverage. Numbers indicate the primer coverage as a percentage of the total sequences of the data base detected by the primer set.*

### Microbial Community Structure in STARs and WTARs

**FIGURE 1:**
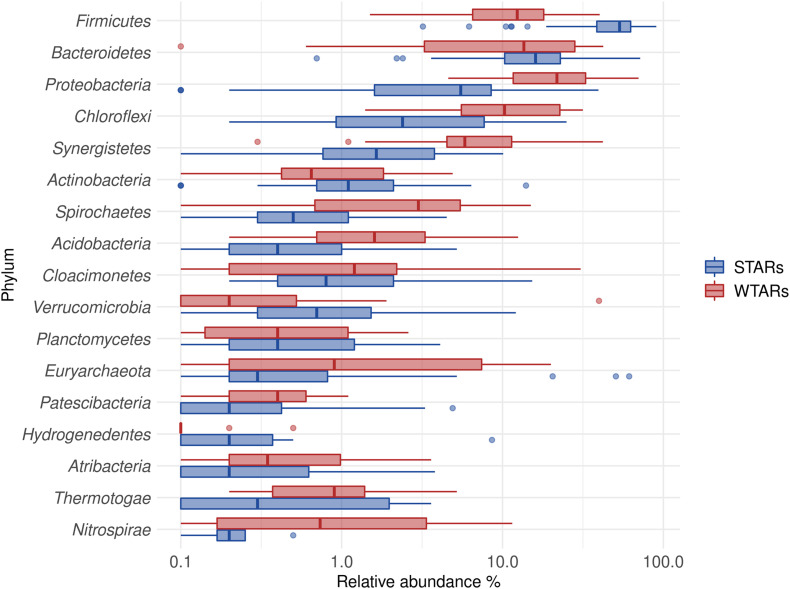
Boxplot of the total community for STARs and WTARs showing the phyla with a relative abundance higher than 2% in at least one reactor.

**FIGURE 2:**
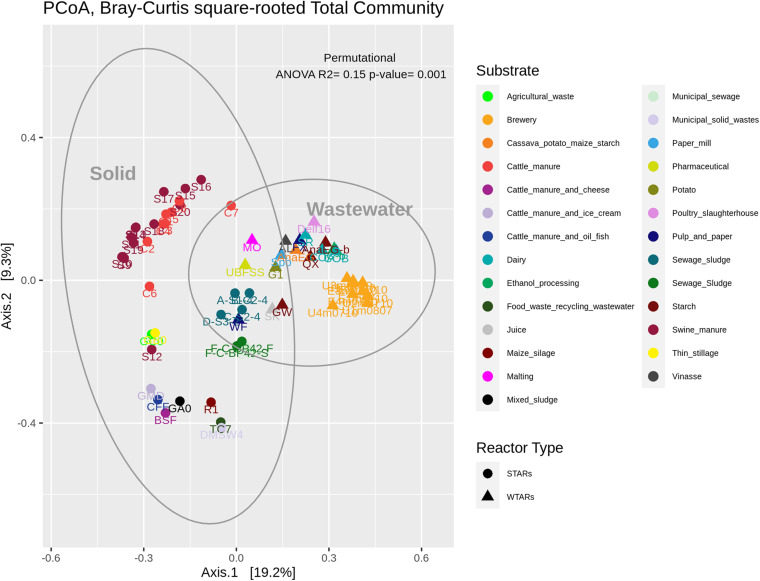
PCoA analysis and PERMANOVA test of the total community. The different colors indicate the different wastes or wastewaters used to fed the reactors. The PERMANOVA test results are shown at the top of the figure (see Table 3).

In total, 1,082,904 high quality sequences from 62 reactors, were retrieved from public databases, ranging from 561 to 164,994 (median of 11,299). All samples were randomly re-sampled at the same depth of 561 sequences before analyzing the data. To be able to compare sequences from different regions, closed-reference OTUs picking method was used. In this method, input sequences are aligned against a reference database. Because input sequences are not compared directly to one another, but rather to an external reference, they do not need to overlap. The closed-reference method allows performing a meta-analysis including sequences derived from different amplification products of the same marker gene. Clustering the pruned and combined data sets from all studies resulted in 13,165 no-singletons OTUs at a similarity threshold of 97%. Bacterial community diversity at phylum level was evaluated for all reactors. Proteobacteria, Chloroflexi, Bacteroidetes, and Firmicutes were the predominant phyla in WTAR and STAR (Figure 1). For WTARs the most abundant phylum was Proteobacteria, while Firmicutes dominated STARs. According to the PCoA analysis, the type of reactor (solid waste vs liquid wastewater treatment) influences the total bacterial community composition. PERMANOVA tests revealed that 15% of the variation of the total community was attributable to the type of reactor (PERMANOVA, *p* < 0.001) (Figure 2 and Table 3).

**TABLE 3:** PERMANOVA (adonis) and multivariate homogeneity of groups dispersions analysis (betadisper) results.

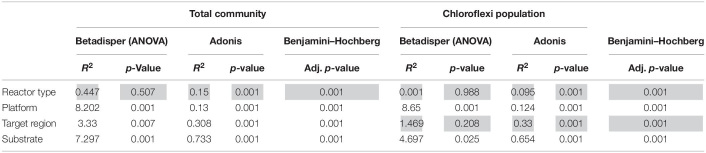

*Groups that satisfy the homogeneity group condition to perform PERMANOVA test are highlighted in gray. *p*-Values were corrected for multiple tests using the Benjamini–Hochberg correction factor. Substrate refers to the feeding according to Table 1.*

Regarding the abundance of the Chloroflexi phylum, we detected higher abundance in WTARs than in STARs. Moreover, Chloroflexi was detected in all reactors studied except for four STARs. In order to determine which variable significantly affected the relative abundance of Chloroflexi, two-way ANOVA analysis was applied. The variables tested were reactor type, primer pair target region, sequencing platform and substrate on the relative abundance of Chloroflexi. Our results revealed that the reactor type had the strongest impact on the abundance of Chloroflexi at phylum level (*R*^2^ = 9.944, *p* < 0.001) followed by the target region (*R*^2^ = 6.045, *p* < 0.001) (Supplementary Table 2).

Interestingly, the hypervariable region V3–V5 recovered the highest relative abundance of the phylum Chloroflexi (median value 7%) followed by V1–V2 (median value 6%) and V5–V9 (median value 0.5%) (Supplementary Figure 1). This is in accordance with the results obtained with the Silva Test Primer analysis (Supplementary Table 3).

### Chloroflexi Community Composition

**FIGURE 3:**
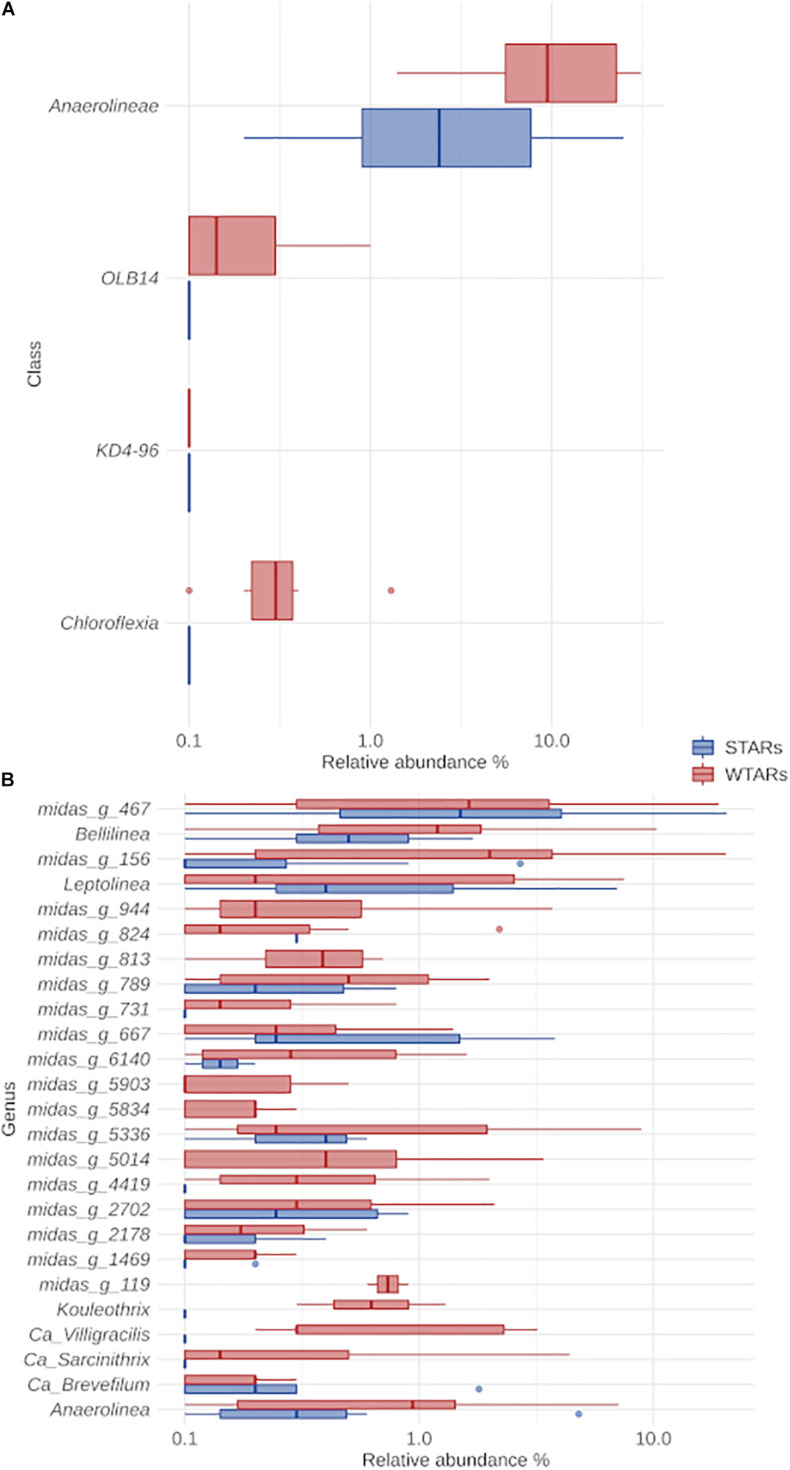
Boxplot of the Chloroflexi phylum for STARs and WTARs showing the relative abundance at: **(A)** class level (showing all the classes present in the systems); **(B)** genus level (showing the 25 most abundant genus which belong to the Anaerolinea class).

The classification of Chloroflexi sequences at the class level showed that Anaerolineae class was predominant in all reactors independently of the substrate, reactor type, 16S rRNA-targeted region and sequencing platform (Figure 3A). The relative abundance of the Anaerolineae in the total community ranged between 1.4 and 31.1% (average: 13.2%) for WTARs and between 0.2 and 24.9% (average: 4.8%) for STARs. The difference in the median relative abundance of Anaerolineae was significantly different when comparing STARs and WTARs (Kruskal–Wallis test *p*-value < 0.001). Other minor classes detected were Chloroflexia and Dehalococcoidia present in some STARs and WTARs.

The diversity of the Chloroflexi community (Shannon index) was significantly higher in WTARs than in STARs (*t*-test, *t* = 2.32, *p* = 0.024) (Supplementary Table 4). The Chao1 and Evenness indices did not show significant differences (Kruskal–Wallis test, *p* > 0.05).

In this meta-analysis, most of the genera detected within the phylum Chloroflexi had no cultured representatives. The recent ecosystem-specific MiDAS 16S rRNA gene amplicon sequencing-based survey, facilitates the understanding of wastewater treatment ecosystem diversity and function (Nierychlo et al., 2019). The advantage of MiDAS 3.6 database is that it provides provisional alphanumeric names at genus and species levels allowing comparisons between unclassified members from different investigations. We found that the four most abundant genera were the same in STARs and WTARs with changes in their relative abundances: midas_g_467, midas_g_156, *Bellilinea* and *Leptolinea* (Figure 3B). While midas_g_467 was abundant in both systems, midas_g_156 was more abundant in WTARs. Despite that both belong to the T78 cluster and are therefore closely related.

**FIGURE 4:**
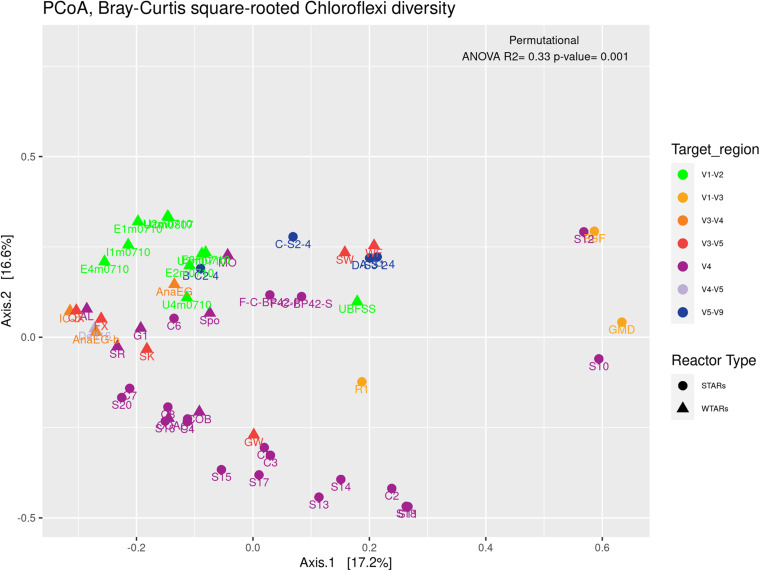
PCoA analysis and PERMANOVA test of the phylum Chloroflexi populations, the different colors indicate the different hypervariable regions. The PERMANOVA test results are shown at the top of the figure (see Table 3).

**FIGURE 5:**
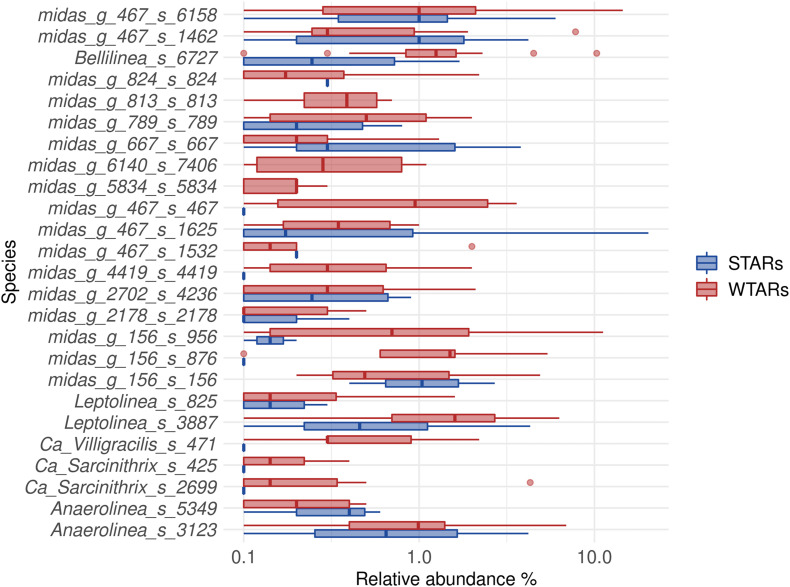
Boxplot of the Chloroflexi phylum for STARs and WTARs showing the relative abundance at species level (25 most abundant).

Principal coordinates analysis and PERMANOVA tests revealed that 33% of the variation within Chloroflexi populations was attributable to the primer target region (PERMANOVA, *p* < 0.001) (Figure 4 and Table 3), while 10% of the variation was attributable to reactor type (Supplementary Figure 2 and Table 3). When performing PERMANOVA test, only with reactors in which the V3–V5 hypervariable region was amplified to avoid the primers bias, 12% of the variation of Chloroflexi populations was attributable to the reactor type (Supplementary Table 2). STARs and WTARs shared 23 of the 25 most abundant OTUs. STARs were dominated by midas_s_6158 (midas_g_467), midas_s_1625 (midas_g_467), midas_s_1462 (midas_g_467), midas_s_3887 (*Leptolinea*), and midas_s_667 (midas_g_667) (Figure 5 and Supplementary Table 5), while WTARs were dominated by midas_s_6158 (midas_g_467), midas_s_956 (midas_g_156), midas_s_6727 (*Bellilinea*), midas_s_3887 (*Leptolinea*), and midas_s_1462 (midas_g_467).

To determine if Chloroflexi populations were shared between reactors or if each reactor type had specific Chloroflexi populations we determined the minimum Chloroflexi core populations. Interestingly, we found that WTARs and STARs showed different Chloroflexi core populations as no OTU was shared by 50% of the reactors (Table 4). Within each reactor type, cores were found. For WTARs the core was composed by 18 OTUs belonging to Anaerolineaceae (midas_g_156, midas_g_467, midas_g_2702, *Leptolinea*, and *Bellilinea* genus) and midas_f_7837 (not assigned at genus level) families, representing at least five genera (Table 4). In STARs the core microbiome was composed by four OTUs belonging to the Anaerolineaceae family (midas_g_467). Only one OTU belonging to the Anaerolineaceae family (midas_g_467) was present in the core of both STARs and WTARs. It should be taken into account that these would be the most abundant OTUs. The low sequencing depth in some samples does not allow to detect OTUs with low abundances.

**TABLE 4:** Minimum core microbiome obtained for STARs and WTARs.

	Accession number	Family	Genera	Species
WTARs	CU922639	Anaerolineaceae	midas_g_156	NA
	EF515722			midas_s_956
	CU922098			
	CU926258		midas_g_467	NA
	CU918857			midas_s_467
	CU921338			midas_s_6158
	CU922464			midas_s_1462
	JQ996679		midas_g_2702	NA
	CU923106		NA	NA
	EF515566		NA	NA
	EF029839		NA	NA
	CU922316		*Leptolinea*	midas_s_3887
	CU921229			
	FQ660222			
	CU923750			
	JQ668574		*Bellilinea*	midas_s_6727
	EF515680			
	FPLM01002501	midas_f_7837	NA	NA
STARs	CU926258	Anaerolineaceae	midas_g_467	NA
	CU923011			midas_s_1462
	AB700375			midas_s_1532
	FJ645708			

*The accessing number of the sequences and the classification at the level of family, genera, and species is shown. NA, not assigned.*

On the other hand, the correlation between Chloroflexi and Euryarchaeota (methanogens) was evaluated using the relative abundances obtained from Li et al. (2015), which included 18 reactors (515F-806R). Interestingly, we found a significant correlation between these two groups (rho: 0.60, *p* < 0.01) (Supplementary Table 6).

### Phylogenetic Analyses of the Sequences Affiliated to the Phylum Chloroflexi

**FIGURE 6:**
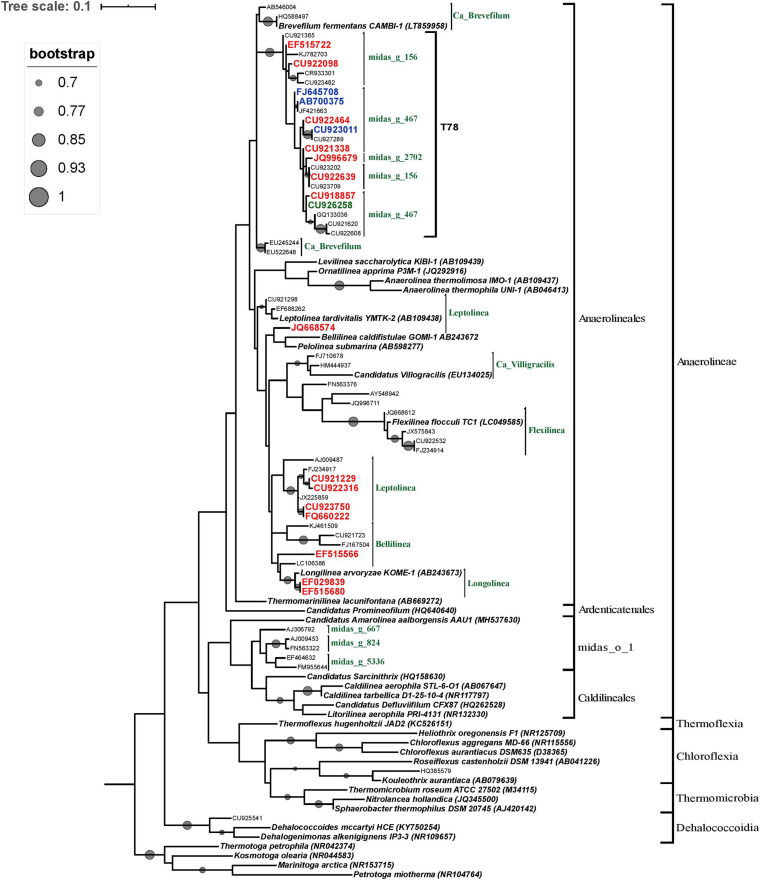
Phylogenetic tree of the phylum Chloroflexi inferred from the 16S rRNA gene sequences. The tree was reconstructed using the ML method and the GTR model. ML bootstrap values greater than or equal to 70% are shown at each node. Bar, 0.01 substitutions per site. The reference OTUs with a relative abundance greater than 5% in at least one reactor are represented by their GenBank accession numbers. Only OTUs from the core microbiome are indicated in color: from STARs are in blue color, from WTARs are in red color. *Thermotoga* spp. sequence served as an outgroup for rooting tree.

In order to determine the phylogenetic position of the most abundant Chloroflexi microorganisms, OTUs with a relative abundance greater than 5% in at least one reactor and OTUs belonging to the minimum core microbiome (defined as Chloroflexi members composed by OTUs shared between 50% of the STARs or WTARs) were used to construct a phylogenetic tree. Most of the selected OTUs sequences were positioned within the order Anaerolineales (52 OTUs) followed by midas_o_1 (5 OTUs). Half of the sequences positioned within the Anaerolineales order clustered with sequences from the T78 genus (22/52 OTUs), including most OTUs sequences from the core microbiome (Figure 6). The genus T78 was recently splitted into midas_g_156 and midas_g_467 (Nierychlo et al., 2020). Within the Anaerolineales order, the 16S ribosomal RNA gene retrieved from the assembled genome of Brevefilum fermentans CAMBI-1 (LT859958) was phylogenetically closely related to the T78 cluster. A few OTUs sequences within Anaerolineales were closely related to 16S rRNA gene sequences from isolated strains or assembled genomes members (Figure 6). The sequences affiliated to the order midas_o_1 had no closely related sequences.

## Discussion

Chloroflexi bacteria are widely distributed on Earth and particularly abundant in engineered systems such as solid waste and wastewater treatment plants. With the aim of better describing the abundance and diversity of this phylum, we performed a meta-analysis of the publicly available diversity datasets (i.e., 16S rRNA metabarcoding) in 62 full scale anaerobic reactors.

### Anaerolineae Class Dominate the Chloroflexi Microbial Communities in the Anaerobic Reactors Studied

Despite the bias generated from using different hypervariable regions we found that the Anaerolineae class largely dominated in both STARs and WTARs which was consistent with previous DNA based surveys (Rivière et al., 2009; Nelson et al., 2011; Bovio et al., 2019). The dominance of Anaerolineae in both types of systems might be explained through several hypotheses. First, most of the isolated strains of Anaerolineae present the potential to degrade cellulose, carbohydrates, and/or proteins anaerobically playing an important role as primary and secondary fermenters, having relevance in the bottlenecking hydrolysis step of anaerobic digestion (Sekiguchi et al., 2001, 2003; Yamada et al., 2005, 2006, 2007a; Narihiro et al., 2015). Thus, the fact that these systems in general treat different fermentable compounds might be favorable for the growth of Anaerolineae.

A second explanation to the predominance of Anaerolineae could be that their growth is significantly stimulated when co-cultivated with methanogens (Sekiguchi et al., 2001, 2003; Yamada et al., 2005, 2006, 2007a; Narihiro et al., 2015) indicating some potential for synergistic relationships. If the Anaerolineae group was a major donor of acetate or hydrogen to methanogens (Narihiro et al., 2015; McIlroy et al., 2017; Zamorano-López et al., 2020), the association in a “spaghetti-like” structure could promote the metabolites transfer flux between both groups, resulting in high methane production rates. In addition, the adhesiveness properties of Methanosaeta (Wiegant, 1986; Angenent et al., 2004; Zheng et al., 2006; Li et al., 2015) and members of Anaerolineae (Xia et al., 2016) could facilitate the metabolites transfer flux improving its dominance in these systems.

Interestingly, Anaerolineae had higher relative abundance in WTARs, which generally have granular biomass, than in STARs, in which the microbial biomass typically does not exhibit granulation but has instead dispersed biomass growth (Hofman-Bang et al., 2003; Klocke et al., 2007). In a previous study, we also determined that UASB reactors with granular biomass presented higher abundance of Anaerolineae than start-up UASB reactors with disperse biomass (Bovio et al., 2019). This might be explained by the fact that the filamentous morphology of Anaerolineae contribute to the granule formation in WTARs (Sekiguchi et al., 2001; Yamada et al., 2005; Satoh et al., 2007) presenting more abundance in reactors with granules than in those with dispersed biomass.

Another explanation could be the fact that the higher flow velocity used in WTARs compared to STARs, may select organisms which can adhere to each other to form well-settling granular sludge, favoring the growth of Anaerolineae in these systems over other microorganisms (Xia et al., 2016).

### Four Genera Within Anaerolineae Predominate in the Full Scale Anaerobic Reactors Studied

We found that the predominant genera and species in STARs and WTARs were the same, but changes in their relative abundances occurred. Four genera were predominant, two of them were closely related to previously described genera (*Bellilinea* and *Leptolinea*) and two of them did not harbor any cultured member and were named by the MIDAS database as midas_g_467 and midas_g_156. As for the isolated members of Anaerolineae, species belonging to the genus *Leptolinea* and *Bellilinea* present filamentous morphology and ferment carbohydrates and proteins (Yamada et al., 2006, 2007a). However, midas_g_467 and midas_g_156 previously named genus T78 in MiDAS database version 2 (Nierychlo et al., 2020), does not have representative cultured members. Despite this, some possible physiological features and ecological roles have been suggested for these genera in anaerobic reactors, which could explain the predominance of genus T78 (Kirkegaard et al., 2017; Zhu et al., 2017; Petriglieri et al., 2018; Jiang et al., 2020). Some of these previous reports suggest that their filamentous morphology and particular position within flocs or granules, might indicate that they play a role in maintaining floc structure (Petriglieri et al., 2018) as well as the granular structure (Zhu et al., 2017). It has also been reported that genus T78 could play an important role in hydrolysis and fermentation steps in the anaerobic digestion process (Yuan et al., 2015; Jiang et al., 2019). T78 also could metabolize alcohols and carbohydrates through syntrophic interactions (Praveckova et al., 2016). In the literature we found that two species belonging to the midas_g_467 correlated positively with different operational parameters: being total ammonia nitrogen, biogas yield, temperature, and organic loading rate (OLR) (Jiang et al., 2020). *B. fermentans* CAMBI-1 (LT859958) was the most closely related metagenome-assembled genome of the T78 cluster. The annotation of this genome suggested that members of the phylotype are important fermenters in mesophilic anaerobic digestion systems (McIlroy et al., 2017). Also, they are co-localized with the filamentous Archaea *Methanosaeta* spp. which suggests a potential undetermined synergistic relationship (McIlroy et al., 2017). Both, the potential fermenting metabolism as well as the potential syntrophism with archaea could explain the dominance of the T78 genus in anaerobic reactors. Our correlation analysis between Euryarchaeota and Chloroflexi supports this hypothesis. Hence, our main hypothesis about the predominance of the same four genera in both systems, relies on their capacity to undergo fermentative and hydrolytic metabolism. Further studies are needed to elucidate the function of these uncultured bacteria, which seem to have different responses to different conditions.

On the other hand, STARs and WTARs showed different core microbiomes of Chloroflexi members. In STARs the Chloroflexi core was less diverse comprising only midas_g_467 genus (4 OTUs) while in WTARs it was composed by at least midas_g_156, midas_g_467, midas_g_2702, *Leptolinea*, and *Bellilinea* genus (18 OTUs). This result was in accordance with the differences observed for the Shannon diversity index. The existence of a core microbiome based on the type of reactor (STARs/WTARs) despite of the heterogeneity of wastewater/solids and environmental factors (e.g., pH, temperature) may suggest that this core could be functionally important in each system. Specific core of microorganism between STARS and WTARs has been detected previously (Calusinska et al., 2018) but regarding Chloroflexi, no previous reports were found. A possible explanation for the different Chloroflexi cores found could be related to the biomass structure found in each type of system. Further exploration of this hypothesis is needed which would contribute to a better understanding of the role of Chloroflexi in the structure of granules and flocs and their metabolic role. Systematic studies examining multiple anaerobic reactor designs with greater depth of coverage and using platforms that generate long-reads or primer-free alternatives should help further define the “core microbiome” of Chloroflexi populations in methanogenic reactors. However, it is important to keep in mind that further recovery of isolated members or interpretative genomes is required to disclose the ecological importance of Anaerolineae as a core population in the anaerobic digestion process.

### Challenges and Limitations of Comparing Sequences Retrieved From Databases Generated With Different Primer Sets and Sequencing Platforms

One of the bottlenecks in comparing microbial profiles is that different studies use different 16S rRNA gene regions generating an important bias. In the present study, we found an underestimation of Chloroflexi populations using primers targeting regions V1–V2 and V5–V9. An example was the underestimation of Anaerolineae class when V1–V2 regions were used recovering only a 5% of the total population. Meanwhile, primers targeting the V3–V5 region in general better reflected the abundances at all taxonomic levels. Previous studies investigating the influence of different hypervariable regions of 16S rRNA gene over the total community in activated sludge identified no perfect region concerning abundant functional genera, except for some regions with less bias, i.e., V1 and V2 (Cai et al., 2013; Guo and Zhang, 2013). However, (Albertsen et al., 2015) recommended primers targeting the V1–V3 regions which better reflected the abundances of Chloroflexi in activated sludge. More studies are needed to identify the best taxonomic profiling effectiveness between different hypervariable regions of the 16S rRNA gene over Chloroflexi populations in anaerobic reactors. As a general rule, drawing conclusions based only on one sequencing region should be avoided due to the potential false negative results, additionally amplicon sequencing of the 16S rRNA gene often limits resolution at genus level. We need primer-free alternatives to get the entire picture of the microbial diversity in anaerobic reactors (Karst et al., 2018). Using the full-length sequence has the potential to become a tool for more precise microbial community profiling that better allows global comparisons of microbiome studies and should potentially increase the accuracy and the resolution of closely related taxa. We highlight the effort to build the MiDAS reference database for microbes in wastewater treatment systems since it allows comparing uncultured microorganisms in different studies (Nierychlo et al., 2020).

Regarding the bias of different sequencing platforms, alpha diversity is significantly affected by both sequence length and depth (Singh et al., 2015). Platforms such as pyrosequencing which were discontinued, had less sequencing depth. To be able to compare samples with different sequencing depths, it is necessary to perform normalization at the lowest value of reads per sample. This leads to a loss of information in the less dominant groups which is an important limitation that has to be taken into account. This problem becomes relevant when comparing data from studies performed in the past (using outdated sequencing platforms) with data obtained more recently where the technology allows a much deeper sequencing. Moreover, it has been reported that Ion Torrent as well as Pyrosequencing platforms had comparatively higher error rates than Illumina platform (Siqueira et al., 2012; Salipante et al., 2014). Although it has been reported that while primer choice considerably influences quantitative abundance estimations, the sequencing platform has relatively minor effects when matched primers are used (Singh et al., 2015). Singh et al. (2015) reported that beta diversity metrics are surprisingly robust to both primer and sequencing platform biases.

New developments in single-cell genomics and metagenomics have in recent years provided new insights into the ecology and evolution of many novel uncultured microorganisms (Albertsen et al., 2013; Sekiguchi et al., 2015; Dam et al., 2020). The genomes have enabled the construction of metabolic models that attempt to explain the physiology of these organisms in detail. The genome-based models form the basis for more extensive investigations, such as *in situ* single-cell characterization, metatranscriptomics, and proteomics (Koch et al., 2014).

### Importance of Standardized Metadata Repositories for Full Scale Methanogenic Reactors

A second issue on performing a meta-analysis is the lack of standardized information on key operational parameters. It is important to have more reactor performance data associated with each reactor, such as compositions of influent waste/wastewater stream, COD loading/removal rate, pH, and temperature. These parameters should be fully used to infer potential roles of lineages of interest and should also be included. In our analysis it was not possible to correlate the microbial community with operational parameters data because in several articles this metadata was incomplete. For this reason, it would be essential to supply the operational parameters together with the raw data in each new study. In this sense, the creation of metadata repositories specific for anaerobic reactors including the 16S rRNA gene datasets must be a great advance. As far as we know there are some initiatives in other ecosystems as the Microbial Antarctic Resource Systems (MARS)^2^ in Antarctic ecosystems.

## Conclusion and Future Directions

From this meta-analysis, we managed to identify that four genera (midas_g_467, midas_g_156, *Bellilinea* and *Leptolinea*) belonging to Anaerolineae class dominated WTARs and STARs with different core populations. All the species observed had no cultured representatives, limiting our knowledge. From the few isolated species belonged from the genera detected, we could hypothesize that the growth of Anaerolineae could be favored by fermentable substrates and by the syntrophic association with methanogenic archaea. In addition, Anaerolineae was more abundant in reactors with granular biomass (WTARs), than in those in which the microbial biomass typically does not exhibit granulation but have instead dispersed biomass growth (STARs), suggesting that Anaerolineae members play an important role in the granule structure due to their filamentous morphology and/or adhesiveness properties. Despite the extensive research that has been done on microbial communities in anaerobic reactors there is no consensus about experimental protocols, bioinformatics analyses or operational data provided, which makes it difficult to perform global comparisons of microbiome studies. We think that more efforts are needed in each study to provide information on key operational parameters associated with each reactor, since it is fundamental to infer potential roles of lineages of interest. Cross-study comparisons can be fruitfully used to understand the complexity of the anaerobic digestion process. The rapidly advancing fields of metagenomics and metatranscriptomics will provide unique insights into the patterns of microbial activity in anaerobic digestion systems, even for species which have not yet been isolated in pure culture.

## Data Availability Statement

The datasets presented in this study can be found in online repositories. The names of the repository/repositories and accession number(s) can be found in the article/Supplementary Material.

## Author Contributions

CE and AC planned the investigation. PB-W performed the bioinformatics analysis. PB-W, AC, and CE discussed the results. PB-W wrote the first draft of the manuscript, and all the authors contributed to manuscript revision and approved the final manuscript.

## Conflict of Interest

The authors declare that the research was conducted in the absence of any commercial or financial relationships that could be construed as a potential conflict of interest.

## Footnotes

1 http://www.bioinformatics.babraham.ac.uk/projects/fastqc

2 http://mars.biodiversity.aq/
