# Supplementary material for: Database Mining to Unravel the Ecology of the Phylum Chloroflexi in Methanogenic Full Scale Bioreactors
Source: Front Microbiol. 2021 Jan 20;11:603234. doi: 10.3389/fmicb.2020.603234 (PMC7854539; doi:10.3389/fmicb.2020.603234)
Supplement: Supplementary file 1 [file Data_Sheet_1.doc]

**Table S1** Studies included in this meta-analysis and operational parameters

| **WTAR** | **Reactor configuration** | **Sample name** | **Substrate** | **Geographical region** | **T(ºC) avg** | **COD removal %** | **Platform** | **Primers** | **Amplicon length** | **Raw data sources** | **Study** |
| --- | --- | --- | --- | --- | --- | --- | --- | --- | --- | --- | --- |
|  | UASB | U1m0807 | Brewery | USA | 27 | 96.5 ± 0.3 | Roche 454 FLX |  | 311 | SRR096048 | Werner et al. 2011 |
|  |  | U2m0710 |  |  | 34 | 96.0 ± 0.5 |  |  |  | SRR095975 |  |
|  |  | U3m0710 |  |  | 29 | 95.4 ± 1.2 |  |  |  | SRR095970 |  |
|  |  | U4m0710 |  |  | 33.7 | 94.1 ± 0.5 |  | 27F AGAGTTTGATCCTGGCTCAG |  | SRR095977 |  |
|  | IC | I1m0710 |  |  | 32 | 90.2 ± 0.9 |  | 338R TGCTGCCTCCCGTAGGAGT |  | SRR095976 |  |
|  | EGSB | E1m0710 |  |  | 32.7 | 88 ± 2 |  |  |  | SRR095972 |  |
|  |  | E2m0710 |  |  | 31.5 | 86 ± 2 |  |  |  | SRR095974 |  |
|  |  | E3m0710 |  |  | 29 | 88.7 ± 1.0 |  |  |  | SRR095971 |  |
|  |  | E4m0710 |  |  | 31 | 94.3 ± 1.1 |  |  |  | SRR095973 |  |
|  | UBF | UBFSS | High-Strength Pharmaceutical | NA | 35 | 60 | Illumina MiSeq | 8F AGAGTTTGATYMTGGCTCAG | 330 | PRJNA331231 | Ma et al. 2017 |
|  |  |  |  |  |  |  |  | 338R TGCTGCCTCCCGTAGGAGT |  |  |  |
|  | UASB | WF | Pulp and paper | Shaanxi (China) | 22 | 68 | Roche 454 GS-FLX |  | 569 | SRR1765717 | Shu et al. 2015 |
|  |  | FX |  |  | 28 | 78 |  |  |  |  |  |
|  |  | GW | Starch |  | 30 | 83 |  | 357F CCTACGGGAGGCAGCAG |  |  |  |
|  |  | QX | Juice |  | 26 | 92 |  | 926R CCGTCAATTCMTTTRAGT |  |  |  |
|  |  | SK | Municipal and campus domestic sewage |  | 24 | 84 |  |  |  |  |  |
|  |  | SW |  |  | 25 | 89 |  |  |  |  |  |
|  |  | G1 | Potato | Netherlands | NA | NA | Illumina MiSeq | 515F GTGCCAGCMGCCGCGGTAA | 291 | PRJNA327299 | Zhu et al. 2016 |
|  |  |  |  |  |  |  |  | 806R GGACTACVSGGGTATCTAAT |  |  |  |
|  |  | Delf16 | Poultry slaughterhouse | Sao Paulo (Brazil) | NA | NA | Ion 318™ | 577F AYTGGGYDTAAAGNG | 347 | ERS1289856 | Delforno et al. 2016 |
|  |  |  |  |  |  |  |  | 924R CCGTCAATTCMTTTRAGT |  |  |  |
|  |  | AL | Vinasse | Uruguay | 20 | 60-78 | Roche 454 FLX |  | 291 | PRJNA453814 | Bovio et al. 2018 |
|  |  | SR | Dairy |  | 20 | 55-90 |  | 515F GTGCCAGCMGCCGCGGTAA |  |  |  |
|  |  | COA | Dairy |  | 25 | 29-97 |  | 806R GGACTACVSGGGTATCTAAT |  |  |  |
|  |  | COB | Dairy |  | 25 | 19-97 |  |  |  |  |  |
|  |  | MO | Malting |  | 20 | 35-80 | Ion Torrent PGM | 520F AYTGGGYDTAAAGNG | 282 |  |  |
|  |  |  |  |  |  |  |  | 802R TACNNGGGTATCTAATCC |  |  |  |
|  |  | Spo | Paper mill | Halden (Norway) | NA | NA | Illumina MiSeq | 515F GTGCCAGCMGCCGCGGTAA | 291 | PRJNA398347 | Sposob et al. 2018 |
|  |  |  |  |  |  |  |  | 806R GGACTACHVGGGTWTCTAAT |  |  |  |
|  | AnaEG | AnaEG | Cassava, potato, maize starch | Hangzhou (China) | NA | NA |  |  | 464 | SRP111625 | Qin et al. 2018 |
|  |  |  |  |  |  |  |  | 341F CCTACGGGAGGCAGCAG |  |  |  |
|  | IC | IC-b | Ethanol processing wastewater | Jilin (China) | 33 | NA |  | 805R GACTACHVGGGTATCTAATCC |  | SRR7281077 | Qin et al. 2019 |
|  | AnaEG | AnaEG-b |  |  | 33 |  |  |  |  | SRR7281078 |  |
| **STAR** |  | DMSW4 | Municipal solid wastes | Madrid (Spain) | 35 | NA | Roche 454 | 27F AGAGTTTGATCCTGGCTCAG | 491 | SRR1928203 | Cardinali-Rezende et al. 2016 |
|  |  |  |  |  |  |  |  | 518R ATTACCGCGGCTGCTGG |  |  |  |
|  | Plug-flow | BSF | Dairy cattle manure suppl. with cheese | Vermont (USA) | 37.8 | - |  |  | 492 | SRR768352 | St-Pierre et al. 2013 |
|  |  | GMD | Cattle manure with ice cream waste |  | 38.3 |  |  | 27F AGAGTTTGATCCTGGCTCAG |  | SRR768352 |  |
|  | Complete mix | CFF | Cattle manure with oil waste |  | 36.1 |  |  | 519R WTTACCGCGGCTGCTGG |  | SRR768352 |  |
|  | CSTR | TC7 | Food waste-recycling wastewater | Gwangju (South Korea) | 58.5 | 79 |  | 787F ATTAGATACCCNGGT | 705 | SRR3281818 | Lee et al. 2016 |
|  |  |  |  |  |  |  |  | 1492R GNTACCTTGTTACGACTT |  |  |  |
|  | - | F-C-BP42-F | Primary and biological sewage sludge | - | 34.6 | 96.54 | Ion Torrent PGM | 515F GTGCCAGCMGCCGCGGTAA | 291 | SRR3198570 | Hao et al. 2016 |
|  | - | F-C-BP42-S |  | - | 34.7 | 96.45 |  | 806R GGACTACHVGGGTWTCTAAT |  | SRR3198571 |  |
|  | - | GA0 | Mixed sludge | Sweden | 38 | - | Illumina MiSeq | 515F GTGCCAGCMGCCGCGGTAA | 290 | SRR6173222 | Liu et al. 2017 |
|  | - | GB0 | Thin stillage |  | 38 | - |  | 805R GACTACHVGGGTATCTAATCC |  | SRR6173226 |  |
|  | - | GC0 | Agricultural waste |  | 38 | - |  |  |  | SRR6173228 |  |
|  | CSTR | R1 | Maize silage | Mecklenburg-Western Pomerania (Germany) | 38 | - | Roche 454 GS | 27F AGAGTTTGATCCTGGCTCAG | 492 | ERR638341 | Lucas et al. 2015 |
|  |  |  |  |  |  |  |  | 519R WTTACCGCGGCTGCTGG |  |  |  |
|  |  | C1 | Cattle manure | Heilongjiang (China) | 32.5 | - | Illumina MiSeq |  | 291 | ERR579094 | Li et al. 2015 |
|  |  | C2 |  | Beijing (China) | 35 | - |  |  |  | ERR579095 |  |
|  |  | C3 |  | Jiangsu (China) | 35 | - |  |  |  | ERR579096 |  |
|  |  | C4 |  |  | 35 | - |  |  |  | ERR579097 |  |
|  |  | C5 |  | Chongqing (China) | 35 | - |  |  |  | ERR579098 |  |
|  | USR | C6 |  |  | 35 | - |  |  |  | ERR579099 |  |
|  | CSTR | C7 |  | Guangxi (China) | 35 | - |  |  |  | ERR579100 |  |
|  |  | C8 |  | Beijing (China) | 35 | - |  |  |  | ERR579101 |  |
|  | USR | S9 | Swine manure |  | 36.5 | - |  |  |  | ERR579102 |  |
|  | CSTR | S10 |  |  | 33 | - |  | 515F GTGCCAGCMGCCGCGGTAA |  | ERR579103 |  |
|  |  | S11 |  | Jiangsu (China) | 35 | - |  | 806R GGACTACHVGGGTWTCTAAT |  | ERR579104 |  |
|  |  | S12 |  | Shanghai (China) | 35 | - |  |  |  | ERR579105 |  |
|  |  | S13 |  |  | 35 | - |  |  |  | ERR579106 |  |
|  |  | S14 |  |  | 35 | - |  |  |  | ERR579107 |  |
|  |  | S15 |  | Zhejiang (China) | 25 | - |  |  |  | ERR579108 |  |
|  |  | S16 |  |  | 25 | - |  |  |  | ERR579109 |  |
|  |  | S17 |  | Ningxia (China) | 35 | - |  |  |  | ERR579110 |  |
|  |  | S18 |  | Sichuan (China) | 36 | - |  |  |  | ERR579111 |  |
|  |  | S19 |  | Guangdong (China) | 30 | - |  |  |  | ERR579112 |  |
|  |  | S20 |  |  | 30 | - |  |  |  | ERR579113 |  |
|  |  | A-S1-4 | Sewage sludge | Seoul (South Korea) | 35 | 45.3 | Roche 454 GS-FLX |  | 705 | SRR3281890 | Shin et al. 2016 |
|  |  | B-C2-4 |  | Daegu (South Korea) | 35 | 39.7 |  | 787F ATTAGATACCCNGGT |  | SRR3281863 |  |
|  |  | C-S2-4 |  | Incheon (South Korea) | 35 | 30.1 |  | 1492R GNTACCTTGTTACGACTT |  | SRR3281959 |  |
|  |  | D-S3-2 |  | Asan city (South Korea) | 35 | 24.3 |  |  |  | SRR3282014 |  |

NA, Not applicable. T (°C), Temperature. COD, Chemical oxygen demand.

Table S2. Statistical analysis to determine the influence of the type of reactor, platform and target region on the relative abundance of phylum Chloroflexi.

|  | Relative abundance of Chloroflexi | | | Chloroflexi population V3-V5 | | | |
| --- | --- | --- | --- | --- | --- | --- | --- |
|  | shapiro-wilk test | two-way ANOVA |  | anova |  | adonis |  |
|  | p-value | R^2^ | p-value | R2 | P-valor | R2 | P-valor |
| Reactor type | 0.06307 | 9.943 | 0.001 | 0.1003 | 0.753 | 0.12269 | 0.001 |
| Platform | 0.06339 | 1.660 | 0.203 | 2.9133 | 0,06561 | 0.12559 | 0.001 |
| Target region | 0.05366 | 6.045 | 0.001 | NA | NA | NA | NA |

NA: not applicable

**Table S3** Relative abundance in percentage of Chloroflexi in STARs and WTARs

| **Target region** | **Primers** | **Chloroflexi (%)** | **Reactor** |  | **Reference** |
| --- | --- | --- | --- | --- | --- |
| V1-V2 | 27F-338R | **6** | U1m0807 | WTAR | Werner et al. 2011 |
|  |  | **7** | U2m0710 |  |  |
|  |  | **5** | U3m0710 |  |  |
|  |  | **2** | U4m0710 |  |  |
|  |  | **6** | I1m0710 |  |  |
|  |  | **6** | E1m0710 |  |  |
|  |  | **7** | E2m0710 |  |  |
|  |  | **3** | E3m0710 |  |  |
|  |  | **5** | E4m0710 |  |  |
|  | 8F-338R | **9** | UBFSS |  | Ma et al. 2017 |
| V3 -V5 | 357F-926R | **30** | FX |  | Shu et al. 2015 |
|  |  | **15** | GW |  |  |
|  |  | **35** | QX |  |  |
|  |  | **16** | SK |  |  |
|  |  | **13** | SW |  |  |
|  |  | **4** | WF |  |  |
| V4 | 515F-806R | **31** | G1 |  | Zhu et al. 2016 |
| V4-V5 | 577F-924R | **24** | Delf16 |  | Delforno et al. 2016 |
| V4 | 520F-802R | **21** | AL |  | Bovio et al. 2019 |
|  |  | **23** | SR |  |  |
|  |  | **33** | COA |  |  |
|  |  | **27** | COB |  |  |
|  |  | **3** | MO |  |  |
| V4 | 515F-806R | **6** | Spo |  | Sposoba et al. 2018 |
| V3-V4 | 341F-805R | **22** | AnaEG |  | Qin et al. 2018 |
|  |  | **13** | IC-b |  | Qin et al. 2019 |
|  |  | **23** | AnaEG-b |  |  |
| V1-V3 | 27F-518R | **0** | DMSW4 | STAR | Cardinali-Rezende et al. 2016 |
| V1-V3 |  | **0** | BSF |  | St-Pierre et al. 2013 |
|  | 27F-519R | **2** | GMD |  |  |
|  |  | **19** | CFF |  |  |
| V5-V9 | 787F-1492R | **0** | TC7 |  | Lee et al. 2016 |
| V4 | 515F-806R | **4** | F-C-BP42-F |  | Hao et al. 2016 |
|  |  | **3** | F-C-BP42-S |  |  |
|  |  | **0** | GA0 |  |  |
| V3-V4 | 341F-805R | **0** | GB0 |  | Liu et al. 2017 |
|  |  | **0** | GC0 |  |  |
| V1-V3 | 27F-519R | **7** | R1 |  | Lucas et al. 2015 |
| V4 | 515F-806R | **7** | C1 |  | Li et al. 2015 |
|  |  | **1** | C2 |  |  |
|  |  | **12** | C3 |  |  |
|  |  | **16** | C4 |  |  |
|  |  | **5** | C5 |  |  |
|  |  | **4** | C6 |  |  |
|  |  | **25** | C7 |  |  |
|  |  | **12** | C8 |  |  |
|  |  | **0** | S9 |  |  |
|  |  | **1** | S10 |  |  |
|  |  | **1** | S11 |  |  |
|  |  | **1** | S12 |  |  |
|  |  | **2** | S13 |  |  |
|  |  | **3** | S14 |  |  |
|  |  | **14** | S15 |  |  |
|  |  | **16** | S16 |  |  |
|  |  | **6** | S17 |  |  |
|  |  | **1** | S18 |  |  |
|  |  | **0** | S19 |  |  |
|  |  | **5** | S20 |  |  |
| V5-V9 | 787F-1492R | **0** | A-S1-4 |  | Shin et al. 2016 |
|  |  | **0** | B-C2-4 |  |  |
|  |  | **2** | C-S2-4 |  |  |
|  |  | **1** | D-S3-2 |  |  |

Table S4. Alpha diversity measures for the Chloroflexi community in WTARs and STARs.

| Reactor | Samples | Shannon H | Evenness | Chao1 |
| --- | --- | --- | --- | --- |
| STARs | C-S2-4 | 2.94 | 0.511 | 49 |
|  | S11 | 1.98 | 0.2785 | 45.5 |
|  | R1 | 0.9193 | 0.209 | 33 |
|  | S12 | 0.89 | 0.4059 | 9 |
|  | CFF | 0.8237 | 0.3256 | 8 |
|  | F-C-BP42-F | 0.4599 | 0.396 | 4.5 |
|  | F-C-BP42-S | 0.2539 | 0.6445 | 2 |
|  | C7 | 3.416 | 0.2145 | 175.5 |
|  | S16 | 3.163 | 0.2686 | 101.6 |
|  | C6 | 3.121 | 0.3022 | 88.15 |
|  | C1 | 2.908 | 0.2544 | 93.43 |
|  | S20 | 2.793 | 0.3473 | 54.2 |
|  | C8 | 2.788 | 0.2083 | 105.6 |
|  | S15 | 2.668 | 0.215 | 97 |
|  | S18 | 2.661 | 0.5727 | 36 |
|  | S17 | 2.614 | 0.2482 | 62.5 |
|  | S13 | 2.443 | 0.3386 | 53.5 |
|  | S14 | 2.336 | 0.2795 | 48.14 |
|  | C5 | 2.282 | 0.3769 | 28 |
|  | C3 | 2.261 | 0.1351 | 117.4 |
|  | D-S3-2 | 2.175 | 0.2751 | 39.5 |
|  | C4 | 2.071 | 0.1134 | 110.6 |
|  | C2 | 1.934 | 0.2767 | 38.2 |
|  | S10 | 1.926 | 0.264 | 42.5 |
|  | GMD | 1.562 | 0.4333 | 12.2 |
| WTARs | Delf16 | 2.76 | 0.1698 | 120.3 |
|  | U4m0710 | 1.82 | 0.617 | 20.5 |
|  | IC-b | 3.929 | 0.2663 | 254.8 |
|  | QX | 3.545 | 0.3808 | 120.1 |
|  | SK | 3.539 | 0.4357 | 107.1 |
|  | AnaEG-b | 3.287 | 0.1537 | 223 |
|  | SW | 3.219 | 0.4999 | 56 |
|  | MO | 2.901 | 0.1856 | 116.4 |
|  | FX | 2.871 | 0.2595 | 89.86 |
|  | WF | 2.827 | 0.5825 | 34.25 |
|  | UBFSS | 2.789 | 0.1849 | 97.55 |
|  | I1m0710 | 2.688 | 0.6127 | 28.67 |
|  | SR | 2.684 | 0.287 | 60.75 |
|  | AnaEG | 2.683 | 0.1393 | 140.1 |
|  | E4m0710 | 2.639 | 0.3889 | 51.17 |
|  | G1 | 2.634 | 0.09349 | 180.1 |
|  | Spo | 2.474 | 0.2525 | 97.6 |
|  | AL | 2.465 | 0.2673 | 53.43 |
|  | U2m0710 | 2.366 | 0.288 | 56.13 |
|  | E1m0710 | 2.354 | 0.5847 | 23.6 |
|  | COB | 2.205 | 0.1711 | 58.5 |
|  | E2m0710 | 2.172 | 0.4874 | 27 |
|  | COA | 2.114 | 0.1533 | 105 |
|  | U3m0710 | 2.042 | 0.6419 | 19 |
|  | U1m0807 | 1.968 | 0.6509 | 13 |
|  | E3m0710 | 1.957 | 0.59 | 26 |
|  | GW | 1.466 | 0.1171 | 52.6 |

**Table S5**. Twenty-five most abundant species of the phylum Chloroflexi

| Accession number | Class | Order | Family | Genus | Species |
| --- | --- | --- | --- | --- | --- |
| CU922464 | Anaerolineae | Anaerolineales | Anaerolineaceae | midas_g_467 | midas_s_1462 |
| AB700375 | Anaerolineae | Anaerolineales | Anaerolineaceae | midas_g_467 | midas_s_1532 |
| CR933301 | Anaerolineae | Anaerolineales | Anaerolineaceae | midas_g_156 | midas_s_156 |
| CU923351 | Anaerolineae | Anaerolineales | Anaerolineaceae | midas_g_467 | midas_s_1625 |
| HQ183891 | Anaerolineae | SBR1031 | midas_f_1469 | midas_g_2178 | midas_s_2178 |
| JQ180422 | Anaerolineae | Caldilineales | Amarolineaceae | Ca_Sarcinithrix | midas_s_2699 |
| EF515732 | Anaerolineae | Anaerolineales | Anaerolineaceae | Anaerolinea | midas_s_3123 |
| CU922316 | Anaerolineae | Anaerolineales | Anaerolineaceae | Leptolinea | midas_s_3887 |
| CU922268 | Anaerolineae | Anaerolineales | Anaerolineaceae | midas_g_2702 | midas_s_4236 |
| FQ659015 | Anaerolineae | Caldilineales | Amarolineaceae | Ca_Sarcinithrix | midas_s_425 |
| CU923743 | Anaerolineae | SBR1031 | midas_f_1469 | midas_g_4419 | midas_s_4419 |
| CU918857 | Anaerolineae | Anaerolineales | Anaerolineaceae | midas_g_467 | midas_s_467 |
| FJ710678 | Anaerolineae | Anaerolineales | Anaerolineaceae | Ca_Villigracilis | midas_s_471 |
| CU923695 | Anaerolineae | Anaerolineales | Anaerolineaceae | Anaerolinea | midas_s_5349 |
| CU924221 | Anaerolineae | RBG-13-54-9 | midas_f_5792 | midas_g_5834 | midas_s_5834 |
| CU921338 | Anaerolineae | Anaerolineales | Anaerolineaceae | midas_g_467 | midas_s_6158 |
| AJ306792 | Anaerolineae | Caldilineales | Amarolineaceae | midas_g_667 | midas_s_667 |
| EF515680 | Anaerolineae | Anaerolineales | Anaerolineaceae | Bellilinea | midas_s_6727 |
| EF515619 | Anaerolineae | Anaerolineales | Anaerolineaceae | midas_g_6140 | midas_s_7406 |
| CU927538 | Anaerolineae | Anaerolineales | Anaerolineaceae | midas_g_789 | midas_s_789 |
| AB514641 | Anaerolineae | midas_o_1 | midas_f_813 | midas_g_813 | midas_s_813 |
| FN563322 | Anaerolineae | midas_o_1 | midas_f_813 | midas_g_824 | midas_s_824 |
| GQ182573 | Anaerolineae | Anaerolineales | Anaerolineaceae | Leptolinea | midas_s_825 |
| CU923482 | Anaerolineae | Anaerolineales | Anaerolineaceae | midas_g_156 | midas_s_876 |
| EF515722 | Anaerolineae | Anaerolineales | Anaerolineaceae | midas_g_156 | midas_s_956 |


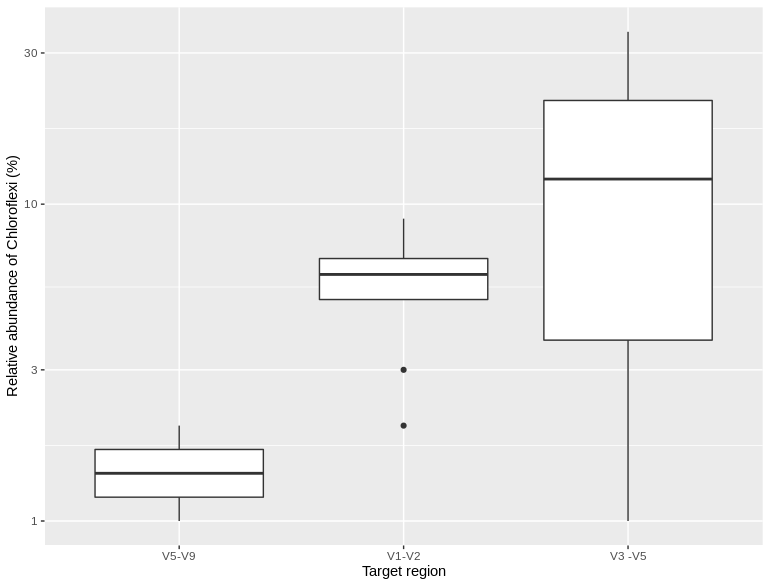


**Fig. S1** Boxplot showing the relative abundance of Chloroflexi using primers targeting for different 16S rRNA gene region (by average).


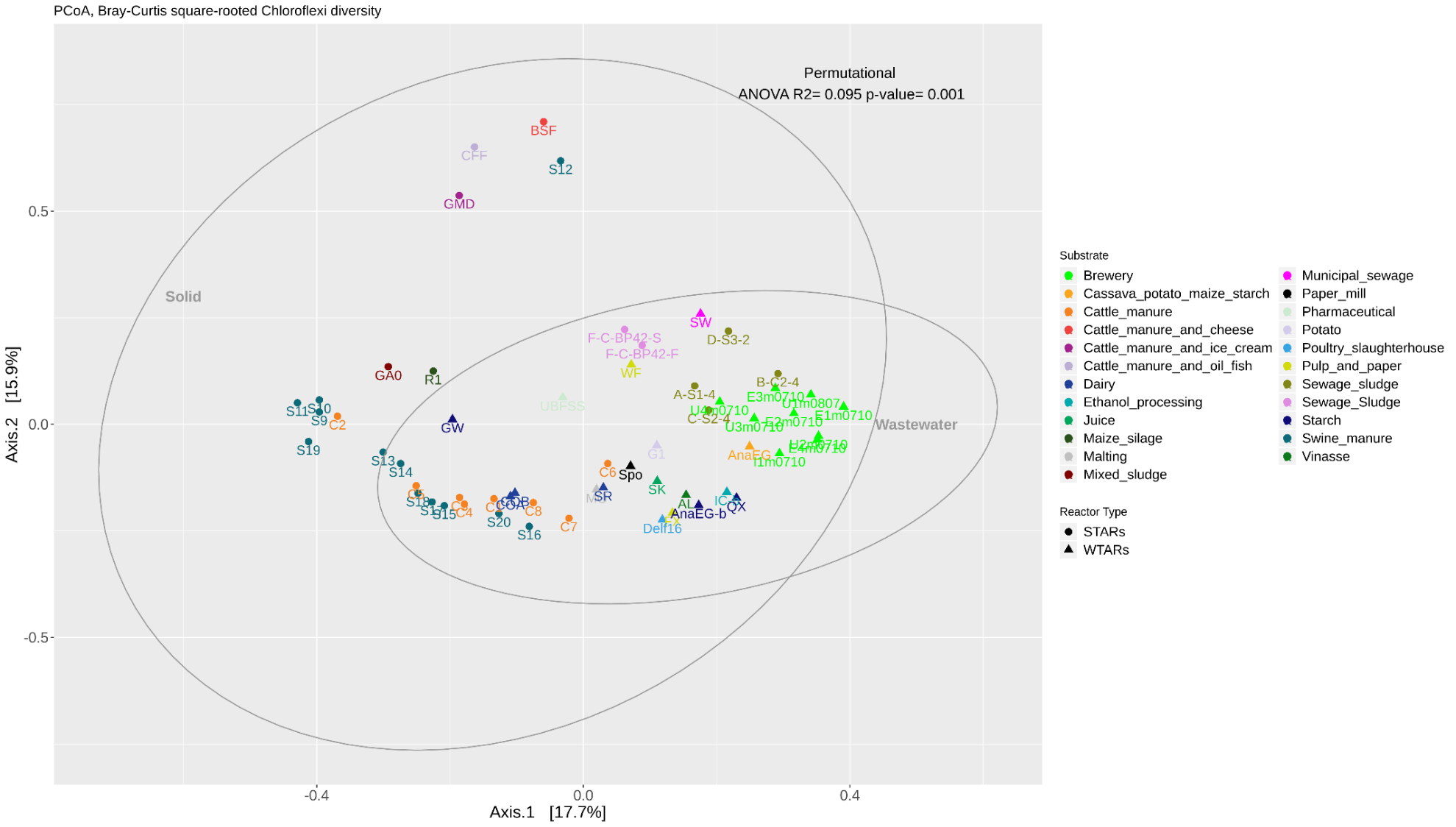


**Fig. S2** PCoA conducted using distance matrices constructed using Bray Curtis metric, PERMANOVA test was used to determine the differences between the samples by reactor type.

Table S6. Correlation analysis between the relative abundance of the phylum Chloroflexi and Euryarchaeota considering different set of primers

|  | Shapiro-Wilk | Spearman | |
| --- | --- | --- | --- |
|  | p-value | rho | p-value |
| All primers | <0.5 | 0.05634082 | 0.6636 |
| 515F-806R 28 reactors | <0.5 | 0.2599891 | 0.1809 |
| 515F-806R 18 reactors  Li et al. 2015 | <0.5 | 0.5969925 | 0.006394 |
